# Early evolution of glial morphology and inflammatory cytokines following hypoxic-ischemic injury in the newborn piglet brain

**DOI:** 10.1038/s41598-022-27034-9

**Published:** 2023-01-06

**Authors:** Elliot J. Teo, Kirat. K. Chand, Stephanie M. Miller, Julie A. Wixey, Paul B. Colditz, S. Tracey. Bjorkman

**Affiliations:** 1 Faculty of Medicine, UQ Centre for Clinical Research, The University of Queensland, Building 71/918 RBWH Herston, Brisbane City, QLD 4029 Australia; 2 Perinatal Research Centre, Royal Brisbane and Women’s Hospital, Herston, QLD Australia

**Keywords:** Neuroscience, Physiology, Stem cells

## Abstract

Neuroinflammation is a hallmark of hypoxic-ischemic injury and can be characterized by the activation of glial cells and the expression of inflammatory cytokines and chemokines. Interleukin (IL)-1β and tumor necrosis factor (TNF)α are among the best-characterized early response cytokines and are often expressed concurrently. Several types of central nervous system cells secrete IL-1β and TNFα, including microglia, astrocytes, and neurons, and these cytokines convey potent pro-inflammatory actions. Chemokines also play a central role in neuroinflammation by controlling inflammatory cell trafficking. Our aim was to characterise the evolution of early neuroinflammation in the neonatal piglet model of hypoxic-ischemic encephalopathy (HIE). Piglets (< 24 h old) were exposed to HI insult, and recovered to 2, 4, 8, 12 or 24H post-insult. Brain tissue from the frontal cortex and basal ganglia was harvested for assessment of glial cell activation profiles and transcription levels of inflammatory markers in HI piglets with comparison to a control group of newborn piglets. Fluorescence microscopy was used to observe microglia, astrocytes, neurons, degenerating neurons and possibly apoptotic cells, and quantitative polymerase chain reaction was used to measure gene expression of several cytokines and chemokines. HI injury was associated with microglial activation and morphological changes to astrocytes at all time points examined. Gene expression analyses of inflammation-related markers revealed significantly higher expression of pro-inflammatory cytokines tumor necrosis factor-α (TNFα) and interleukin 1 beta (IL-1β), chemokines cxc-chemokine motif ligand (CXCL)8 and CXCL10, and anti-inflammatory cytokine transforming growth factor (TGF)β in every HI group, with some region-specific differences noted. No significant difference was observed in the level of C-X-C chemokine receptor (CCR)5 over time. This high degree of neuroinflammation was associated with a reduction in the number of neurons in piglets at 12H and 24H in the frontal cortex, and the putamen at 12H. This reduction of neurons was not associated with increased numbers of degenerating neurons or potentially apoptotic cells. HI injury triggered a robust early neuroinflammatory response associated with a reduction in neurons in cortical and subcortical regions in our piglet model of HIE. This neuroinflammatory response may be targeted using novel therapeutics to reduce neuropathology in our piglet model of neonatal HIE.

## Introduction

Neuroinfammation is one of the hallmarks of hypoxic-ischemic encephalopathy (HIE)^1^. The molecular signals that result from acute cell death during the primary insult have a modulatory effect on glial cell gene expression, placing astrocytes and microglia in a so-called activated state^2^. Activated astrocytes and microglia are identified morphologically by enlargement of the cell body, hypertrophy of the primary processes and loss of tertiary arborization. Activated glial cells release a cocktail of cytokines, chemokines, and other molecules to drive this inflammatory response further^2^. Early-response interleukin (IL)-1β and tumor necrosis factor (TNF)α cytokines are believed to be influential in the progression of HIE in the neonatal brain by promoting the synthesis of other cytokines, adhesion molecules and chemokines such as C-X-C motif chemokine ligand (CXCL)8 and CXCL10. Furthermore, chemokines CXCL8 and CXCL10 draw peripheral macrophages into the brain and local microglia towards areas of focal damage, thus exacerbating the local injury.

While it is generally accepted that neuroinflammation is present following acute HI, the temporal dynamics of this process have rarely been investigated in large animal models*,* and even less data exists of inflammation during the latent phase of injury (0–6 h post-HI)^3^. Upregulations of TNFα, IL-1β and IL-6 cytokines in the serum, plasma and cerebrospinal fluid (CSF) have been observed in neonates following HIE and is related to the severity of the disorder and outcome^4–6^. Most of the research has studied human serum cytokine levels at one or two time points, generally between 24 and 72 h. Animal studies allow for frequent serial sampling at multiple timepoints. Woods et al.^7^ in the nonhuman primate model measured plasma cytokines, chemokines, and growth factors at 3, 6, 24, 72, and 96 h after umbilical cord occlusion, treated with hypothermia therapy (HTH) and erythropoietin and observed complex alterations in circulating chemokines based on severity of injury and response to therapy with hypothermia and erythropoietin. Rocha-Ferreira et al., reported alterations in serum levels of a number of cytokines following hypoxic-ischemic (HI) injury and HTH in a neonatal pig model^8^. Rocha-Ferreira et al. showed that cytokines may have a variety of temporal profiles after HI, and most importantly, they did not correlate with CSF levels^8^. Therefore, serum cytokine levels are not a suitable measure of brain inflammation and neuroinflammation should be directly measured.

To date, there is little published data evaluating the evolution of neuroinflammation in the brain of large animal models of neonatal HIE. Our aim was to observe the evolution of the neuroinflammatory response in our piglet model in the first 24 h following a neonatal HI insult. We hypothesized that HI injury will be associated with the activation of microglia and astrocytes, with concurrent changes in expression of inflammatory markers TNFα, IL-1β, CXCL8, CXCL10, CCR5 and TGF-β.

## Methods

### Animals

**Figure 1:**
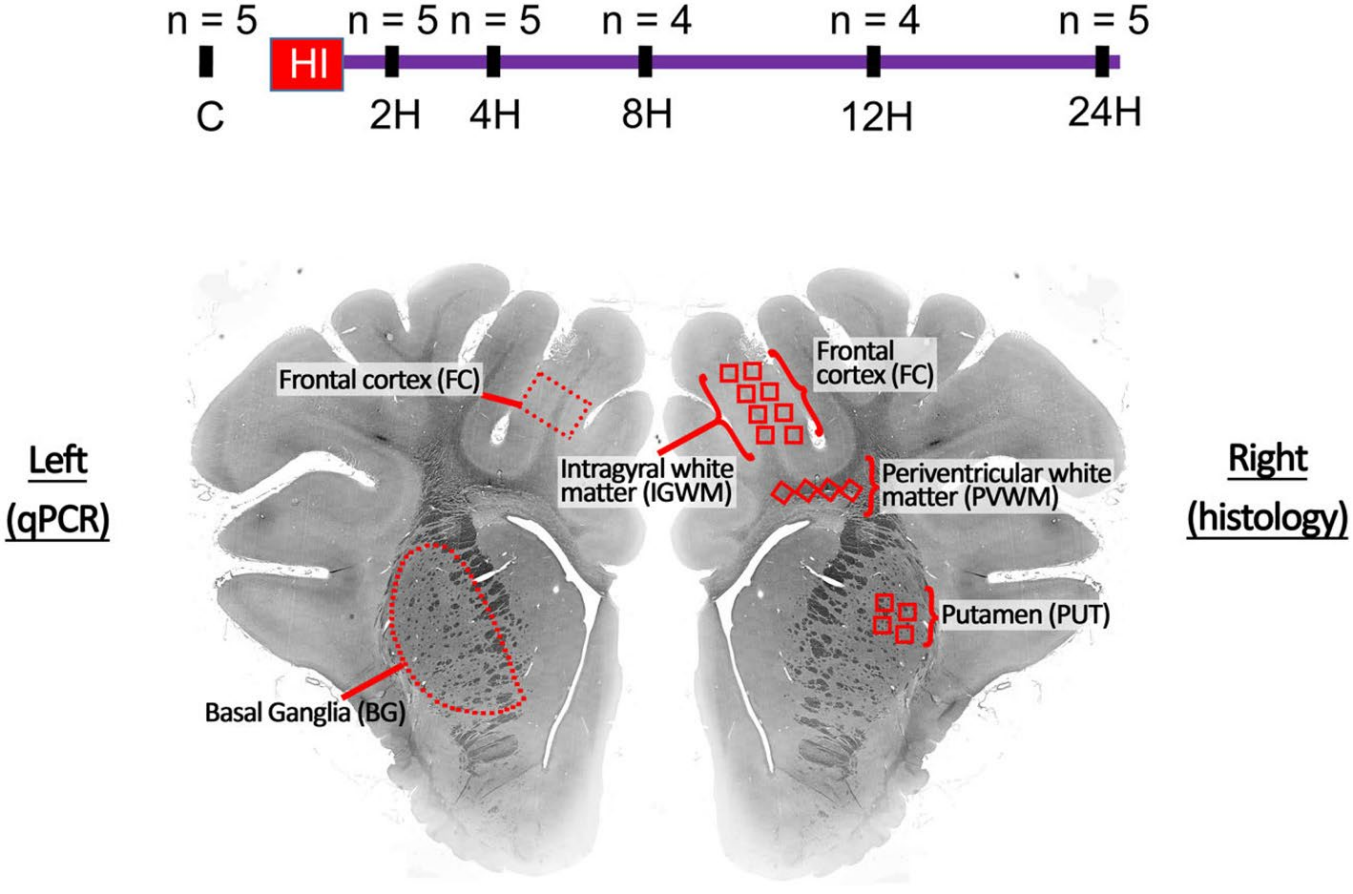
Timeline of the experimental protocol and brain regions of interest. Dark dashes indicate a cull point. The red region indicates the 30–45-min period of hypoxic-ischemic insult. The lower panel indicates the approximate regions of interest for qPCR (left) and histological analysis (right).*BG* basal ganglia; *FC* frontal cortex; (number)*H* group culled hours post injury; *HI* Hypoxic-ischemic; *n* number of animals per experimental group; *IGWM* intragyral white matter; *PVWM* periventricular white matter; *PUT* putamen; *qPCR* quantitative polymerase chain reaction.

Experiments were performed and reported in accordance with the animal research: reporting of in vivo experiments (ARRIVE) Essential 10 guidelines (See Supplementary File). Approval for this study was obtained from the University of Queensland Animal Experimentation Ethics Committee and carried out following the National Health and Medical Research Council guidelines (Australia) (UQCCR/224/16/NHMRC). Twenty-eight newborn Large White piglets (< 24 h old) with an average weight of 1.77 kg ± 0.23 kg were obtained from the University of Queensland Gatton piggery. Animals of both sexes were allocated to either the postnatal day 1 (C) control group (n = 5) or one of the five hypoxic-ischemic injured groups culled at different timepoints following HI: 2 h (2H; n = 5), 4 h (4H; n = 5), 8 h (8H; n = 4), 12 h (12H; n = 4), and 24 h (24H; n = 5). These animals were subjected to our HI protocol and culled at their defined timepoint, where their brains were removed and regions of interest were prepared for histological and gene expression analysis as illustrated in Fig. 1.

### Experimental setup

Piglets were sedated with 2–4% isoflurane (Bayer, Pymble, NSW, AUS) via a facemask and a Goldman Vaporizer. Piglets were then anesthetized with a 5 mg/kg propofol (Diprivan 1%, AstraZeneca Pty Ltd., NSW, AUS) injection through a cannulated (24 gauge) ear vein. Intravenous anesthesia was maintained with propofol (9 mg/mL)/alfentanil (50 µg/mL) (Rapifen, ICI Pharmaceuticals, VIC, AUS) throughout the experiment. Anesthesia was delivered at a rate of 10 mg/kg/h until intubation with a size 3.0 or 3.5 cuffed endotracheal tube. Anesthesia was then increased to 20 mg/kg/h for 15 min, reduced to 15 mg/kg/h for 15 min, and reduced again to 10 mg/kg/h for the duration of the HI insult. Piglets were ventilated using an SLE Newborn 250 ventilator (Surrey, United Kingdom) or a Hamilton-C1 neo ventilator (Bonduz, Switzerland). Ventilation rate was set to 30 breaths per minute, a fraction of inspired oxygen (FiO_2_) of 21%, peak inspiratory pressure of 12 cm H_2_O, positive end-expiratory pressure of 5 cm H_2_O, and inspiratory time ratio of 0.74. Ventilation pressures and FiO_2_ were titrated to maintain end-tidal CO_2_ (ETCO_2_) between 35 and 45 mmHg and oxygen saturation (SO_2_) of > 95%. Physiological monitoring of SO_2_, ETCO_2_, rectal temperature, heart rate, and blood pressure were performed using a Marquette Tramscope 12C (Medical Systems, WI, USA). Arterial blood gas of pH, arterial base excess (ABE), lactate, pCO_2_, pO_2_, bicarbonate, hemoglobin, and glucose were measured before, during, and after the HI insult (ABL800, Radiometer, VIC, AUS). Following the HI insult, anesthesia was adjusted between 4 and 12 mg/kg/h to maintain sedation until euthanasia.

### Hypoxic-ischemic insult protocol

Piglets were stabilized before the HI insult for a period of 90–120 min following anesthesia induction. FiO_2_ was reduced from 21(air) to 4% to induce hypoxia and titrated between 210% to maintain amplitude-integrated EEG (aEEG) < 5 μV, and approximately 10 min of MABP < 30 mmHg. This has been measured to be the lower limit of cerebral autoregulation in the piglet, and defines our ischemic period^9^. The HI insult was terminated by the return of FiO_2_ to 21%. This HI protocol is well established by our laboratory^10–19^ and has previously given a consistent, clinically relevant injury.

### Euthanasia and post-mortem

At the assigned euthanasia time-point, animals were sedated with 1–2% inhalational isoflurane (Bayer, NSW, AUS) and administered an overdose of sodium pentobarbitone (325 mg/ml) (Lethabarb 120 mg/kg, intraperitoneal). Brains were flushed with saline and removed from the skull, coronally sliced at 3 mm intervals and hemisected. The left hemisphere was separated into regions of interest and snap-frozen in liquid nitrogen. All slices from the right hemisphere were immersion fixed in 4% paraformaldehyde, 0.1 M phosphate-buffered saline (PBS, pH 7.4) overnight (18–20 h at 4 °C), then transferred to 0.5% paraformaldehyde in 0.1 M PBS and 0.5% sodium azide. The heart, lungs, kidneys, liver, and spleen were weighed and inspected for gross pathology.

### Histology

Slices from the right hemisphere were processed through graded alcohols and xylenes for 20 h using the HistoCore PEARL (Leica, USA). Processed slices were embedded in paraffin wax using the HistoCore arcadia H (Leica, USA). Tissue blocks were cut in 6 µm serial sections using a Leica microtome and mounted on Menzel Gläser Superfrost Plus (Menzel, Germany) microscope slides.

### Fluoro-Jade C (FJC)

Degenerating neurons were stained using Fluoro-Jade C (FJC). Sections were dewaxed in xylene and rehydrated through graded alcohols using an automated system (Leica ST5010 Autostainer XL, Leica Biosystems North Ryde, NSW, Australia). Dewaxed and rehydrated slides were incubated in 0.006% potassium permanganate solution for 5 min. Slides were rinsed in PBS for 2 min then transferred to a 0.0001% solution of FJC (Merck Millipore, Germany) dissolved in 0.1% acetic acid vehicle, containing 4',6-Diamidino-2-Phenylindole (DAPI) to counterstain the nuclei for 15 min. Slides were washed 3 × 5 min in PBS followed by air drying on a slide warmer at 50 °C for 5 min. Slides were cleared with xylene for 1 min before being cover slipped with DPX mounting media (Sigma-Aldrich).

### Immunofluorescence

Dewaxed and rehydrated slides underwent heat-induced antigen retrieval was performed in a decloaking chamber (90 °C 20 min, Biocare Medical). A hydrophobic barrier was drawn around the tissue using a PAP pen (Abcam; ab2601) followed by non-specific blocking with 5% donkey serum (Sigma–Aldrich; D9663) in PBS with 0.5% Triton-X 100.

Sigma–Aldrich; 9036-19-5) and 0.05% tween-20 (Sigma–Aldrich, 9005-64-5) for 1 h at room temperature (RT). Primary antibodies (neuronal nuclei (NeuN), glial fibrillary acidic protein (GFAP), ionized calcium binding protein a(Iba)-1, TNFα, IL-1β, cleaved caspase 3(C-cas3), cluster of differentiation (CD)-34) were incubated according to optimized conditions (See Additional File 1. Table 1). Slides were washed in PBS followed by incubation with species-specific secondary fluorophores (See Additional File 1. Table 2) at RT for 1 h. Secondary only (negative) controls were also run to rule out non-specific binding (data not shown). A Zeiss Axio Microscope (Axioscope 5; Zeiss Microscopy, Australia) with the Plan-Apochromat 10x/0.45 M27 objective lens (878.94 μm × 662.84 μm), the EC Plan-Neofluar 20x/0.50 M27 objective lens (439.47 μm × 331.42 μm), or EC Plan-Neofluar 40x/0.75 M27 objective lens (219.74 μm × 165.71 μm) were used to visualize stained and labelled sides. Photomicrographs were captured with an Axiocam503 camera (Zeiss Microscopy, Australia). All images were processed with Adobe Photoshop. Immunofluorescence images were pre-processed using the subtract background tool using a “Rolling Ball Radius” of 50 pixels. Fiji ImageJ version 2.3.0^20^ https://imagej.nih.gov/ij/download.html with the Cell Counter plugin was used to quantify positively labelled cells. Cell counts were performed in four fields from each region per animal in triplicate. GFAP labelling area % was quantified using Image J software version 2.3.0^20^ https://imagej.nih.gov/ij/download.html. Pixels with intensity values approximately like the background were replaced with the mean background intensity value using the subtract background tool with the “Rolling Ball Radius” equal to 50. A label mask was created using the adjust threshold tool and the Otsu algorithm. Finally, the area of the immunolabelling was divided by the total area of the region of interest imaged to obtain the area %. Cell counting and measurement of area coverage were performed in the frontal cortex (FC), periventricular white matter (PVWM), intragyral white matter (IGWM) and putamen (PUT) by a blinded observer. These regions were chosen as they are within the watershed zones and are vulnerable to HI injury^21–23^.

### Gene expression analyses

#### Total RNA extractions

Snap frozen brain tissue (frontal cortex and basal ganglia) (~ 30 mg) was disrupted using a needle and syringe. Total RNA was extracted using a Qiagen RNeasy (Scientifix, VIC, AUS), according to the manufacturer's instructions. RNA yield and quality were determined using a NanoDrop spectrophotometer (NanoDrop ND-1000 system, Thermo Fischer Scientific). A 260/280 ratio of ~ 2.0, and a 260/230 ratio 2 > 2.2 was accepted for RNA as per Thermo Scientific Technical Bulletin T042.

#### cDNA synthesis

A reverse transcription kit (SuperScript III First-Strand System for RT-PCR; Invitrogen cat#18080-051) was used for cDNA synthesis. cDNA was synthesized from 1 μg of RNA with SuperScript III (Life Technologies, VIC, AUS), using random hexamers. cDNA yield and quality were determined using a NanoDrop spectrophotometer (NanoDrop ND-1000 system, Thermo Fischer Scientific). A 260/280 ratio of ~ 1.8 and a 260/230 ratio 2 > 2.2 was accepted for DNA as per Thermo Scientific Technical Bulletin T042.

#### Quantitative polymerase chain reaction

Quantitative polymerase chain reaction (qPCR) was performed using a Rotor-Gene 6000 (Qiagen, VIC, AUS). The total reaction volume was 20 µL, containing 100 ng cDNA, 10 µL SYBR green PCR master mix (Invitrogen, QLD, AUS), and 2 μM each of forward and reverse gene-specific primers (Sigma–Aldrich, NSW, AUS; See Additional File 1. Table 3). Reactions were incubated for 10 min at 95 °C, followed by 40 amplification cycles (20 s—95 °C, 30 s—55–65 °C (annealing—primer specific), and 40 s—72 °C). No-template and no-SYBR green controls were included in every run. Results were normalized to glyceraldehyde 3-phosphate dehydrogenase (GAPDH). Data were analyzed using the ΔΔCT method ^24^ and presented as log fold changes (log_2_^ΔΔCT^) and 95%CI [upper, lower].

### Statistical analyses

Sample size calculation was not performed as this project utilised archived tissue that had already been collected for the cohort of animals. The statistical analyses were conducted using R^25^ https://cran.r-project.org/bin/windows/base/, with the tidyverse package^26^ or GraphPad Prism version 9.0.2 for Windows, GraphPad Software, La Jolla California USA, www.graphpad.com. Initial data exploration with Shapiro–Wilks tests for normality and Levene's test for the assumption of equal variance. Where no significant departures from the assumption normality or equal variance were found, ordinary one-way analysis of variance was used. Where statistically significant differences were found between groups, the two-stage linear step-up procedure of Benjamini, Krieger and Yekutieli was used to determine where these differences lay. These data are presented as mean ± standard deviation. FJC cell counts exhibited non-normal distribution that could not be adequately corrected. FJC^+^ cell counts also exhibited heteroskedasticity and were evaluated using Kruskal–Wallis tests with the two-stage linear step-up procedure of Benjamini, Krieger Yekutieli. For clarity, the results of the ANOVA and Kruskal Wallis tests are denoted ‘*p*’, and the results of the two-stage linear step-up procedure are denoted with ‘q’ value in keeping with the original notation^27^. Pearson correlation analyses were performed between inflammatory cytokine gene expression and the numbers of mature neurons, where a significant linear correlation was observed, a linear regression was performed and model fit to the data. Statistical significance was accepted when *q* ≤ *0.05,* or *P* ≤ *0.05* where appropriate*.* Descriptive statistics of these data are presented as median with 95% confidence intervals [lower bound, upper bound]. Data analyses were conducted under blinded conditions for all measures.

### Ethics approval and consent to participate

Approval for this study was obtained from the University of Queensland Animal Experimentation Ethics Committee and carried out following the National Health and Medical Research Council guidelines (Australia) (UQCCR/224/16/NHMRC).

## Results

### Hypoxic-ischemic insult

Data were collected from 28 animals for this study, with two females and three males in the control group, 2H, 4H, 24H; and two females and two males in 8H and 12H groups. There were no significant differences in body weight [F (5,22) = 0.3768, *p* = *0.8592*] and no observed instances of overt systemic organ pathology at post-mortem. Electrographic seizures were observed in one animal in the 12H group. Physiological data for baseline and end insult parameters for the HI injured animals are shown in Table 1. No significant differences were observed between any of the HI injured groups on any measured physiological parameter pre or immediately post-insult.

**Table 1:** Group HI injury outcomes.

	2H (n = 5)	4H (n = 5)	8H (n = 4)	12H (n = 4)	24H (n = 5)	F value	*P* value
Total hypoxia duration (mins)	30.5 ± 3.0	31.85 ± 5.4	29.8 ± 4.6	30.8 ± 3.6	31.8 ± 2.4	0.21	0.92
MABP pre insult (mmHg)	44 ± 7	40 ± 6	39 ± 5	40 ± 11	40 ± 3	0.34	0.84
MABP max (mmHg)	62 ± 10	59 ± 9	62 ± 14	64 ± 22	62 ± 15	0.08	0.98
MABP end insult (mmHg)	17 ± 5	14 ± 4	19 ± 5	17 ± 7	15 ± 3	0.65	0.62
Total ischemic period (mins)	11.7 ± 5.9	15.5 ± 7.03	12.6 ± 9.7	14.6 ± 11.2	16.9 ± 7.37	0.32	0.85
Time bradycardic (mins)	2.0 ± 2.2	2.2 ± 2.6	4.1 ± 4.8	4.7 ± 4.9	1.42 ± 1.25	0.84	0.51
pH before insult	7.466 ± 0.066	7.446 ± 0.101	7.461 ± 0.034	7.439 ± 0.049	7.419 ± 0.022	0.42	0.78
pH end insult	7.105 ± 0.149	7.111 ± 0.047	7.044 ± 0.061	7.041 ± 0.028	7.081 ± 0.059	0.69	0.60
HCO_3_^-^ pre insult (mmol/L)	29.6 ± 4.2	28.2 ± 6.5	28.0 ± 0.2	25.4 ± 2.4	26.7 ± 1.1	0.65	0.65
HCO_3_^−^ end insult (mmol/L)	12.9 ± 5.0	13.6 ± 2.8	11.6 ± 2.2	10.6 ± 2.7	10.6 ± 1.1	0.93	0.46
ABE before insult (mmol/L)	5.5 ± 4.5	3.9 ± 7.0	4.2 ± 0.3	1.1 ± 1.3	2.1 ± 1.1	0.83	0.51
ABE end insult (mmol/L)	− 13.7 ± 7.5	− 14.4 ± 3.4	− 15.7 ± 3.3	− 17.3 ± 4.3	− 17.0 ± 1.7	0.55	0.70
pCO_2_ before insult (mmHg)	38.9 ± 3.6	40.6 ± 2.9	39.9 ± 4.1	37.2 ± 2.2	41.4 ± 3.3	1.12	0.37
pCO_2_ end insult (mmHg)	46.9 ± 8.4	46.8 ± 9.8	54.9 ± 6.3	47.8 ± 13.7	41.2 ± 4.2	1.33	0.29
pO_2_ pre insult (mmHg)	99.6 ± 20.8	104.7 ± 29.2	131.8 ± 2.6	100.7 ± 15.0	94.6 ± 3.7	2.78	0.06
pO_2_ end insult (mmHg)	14.1 ± 2.6	21.9 ± 8.4	24.1 ± 7.2	19.4 ± 9.3	20.0 ± 5.0	1.08	0.39
Glucose pre insult (mmol/L)	6.7 ± 2.1	5.9 ± 1.4	7.5 ± 2.2	5.3 ± 1.6	5.6 ± 1.0	1.40	0.27
Glucose end insult (mmol/L)	8.2 ± 2.0	5.3 ± 2.4	8.6 ± 2.0	7.4 ± 2.9	7.2 ± 1.3	1.56	0.23
Lactate pre insult (mmol/L)	1.2 ± 0.5	1.7 ± 0.3	1.9 ± 0.4	2.3 ± 0.3	1.7 ± 0.7	1.43	0.26
Lactate end insult (mmol/L)	8.1 ± 6.0	9.7 ± 4.5	5.2 ± 1.6	5.2 ± 1.8	7.4 ± 2.9	0.74	0.58

No significant differences were observed between groups at pre or post insult times. Statistical comparisons refer to results of ordinary one-way ANOVA. Statistical significance was accepted at *p* < 0.05.

*ABE* arterial base excess; (number)*H* group culled hours post injury; *MABP* Mean arterial blood pressure; *mmHg* millimetres of mercury; *mmol/L* millimoles per liter; *pCO*_*2*_ partial pressure of carbon dioxide; *pO*_*2*_ partial pressure of oxygen.

### Hypoxic-ischemic injury is associated with sustained activation of microglia in the first 24 h

**Figure 2:**
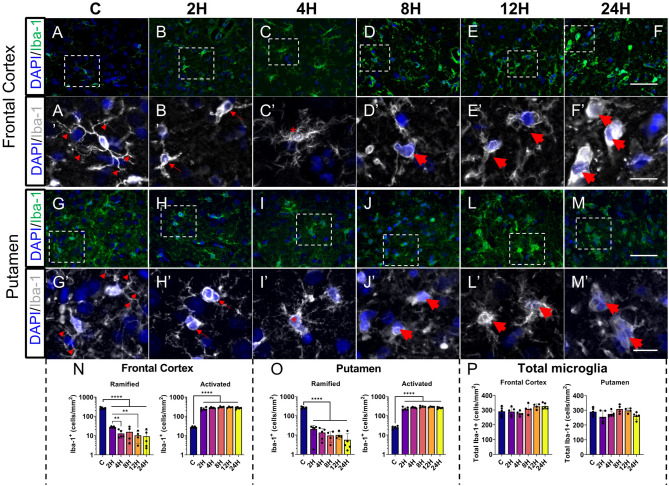
Hypoxic-ischemic insult is associated with increased microglial activation in cortical and subcortical regions of interest. (**A**–**F**) Representative images of Iba-1 expression in the frontal cortex (FC) piglet brains. (A’–F’, G’–M’) Illustrate the typical morphology of microglia observed in the respective groups. Control I group microglia displayed a resting state with long branching processes (A’, G’; triangles). In the 2H group, cell bodies were relatively similar, though processes were much shorter’(B’ H’; thin tailed arrows). Piglets in the 4H group had microglia that were bushy in appearance and covered a much smaller area than the C group’(C’ I’; marked with an asterisk). In 8H, 12H, and 24H groups, the Iba-1 + cells were round and appeared larger (D’–F’ J’ M’; thick-tailed arrows). Morphological classification revealed significantly more activated microglia in the frontal cortex (**N**) and putamen (**O**) with fewer resting microglia. There was a significant difference in the total number of microglia in the frontal cortex, but no differences between groups were significant at the post-hoc level, no significant difference was observed in the putamen (**P**). Individual data points are the average aggregation of 4 separate fields from 3 technical replicates. Columns illustrate the mean with error bars at one standard deviation. Statistical comparisons refer to ordinary one-way analysis of variance test with a two-stage linear step-up procedure of Benjamini, Krieger and Yekutieli. Statistical significance was accepted at q < 0.05 (^∗∗^q < 0.01, ^∗∗∗∗^q < 0.0001). High magnification image scale bar = 25 μm; low magnification image scale bar = 100 μm. *C* control group; *FC* frontal cortex; (number)*H* group culled hours post injury; *Iba-1* ionized calcium binding adaptor molecule 1; *PUT* putamen.

Iba-1 was used to visualize microglia in the frontal cortex (FC) and the putamen (PUT) (Fig. 2). In total, 58426 microglia were classified in the FC, and 43556 microglia in the PUT. Resting (ramified) microglia were characterized by round or oval cell bodies with fine extended processes, and activated microglia by more intense cell bodies and thickened retracted processes. Control group microglia displayed long fine processes with clear tertiary branching (Fig. 2 A’, G’; arrowheads) In the 2H group cell bodies were relatively similar, though processes were much shorter (Fig. 2 B’, H’; thin tailed arrows), than those in the C group. Piglets in the 4H group had microglia that were bushy in appearance and covered a much smaller area than the control group (Fig. 2 C’, I’; marked with asterisk). In 8H, 12H, and 24H groups the Iba-1 + cells were round and appeared larger (Fig. 2 D’–F’, J’–M’; thick tailed arrows). When microglia were classified into resting state or activated, there were significant differences in the numbers of resting microglia in the frontal cortex (Fig. 2 N; FC, F (5, 6.480) = 244.4, *p* < 0.0001). Post-hoc analyses found the numbers of resting microglia in the frontal cortex were significantly fewer at all timepoints (2H, 27 ± 3; 4H, 13 ± 6; 8H, 16 ± 12; 12H, 10 ± 6; 24H, 9 ± 8) compared with the control group (264 ± 31, q < 0.001 for all comparisons). Further differences were observed between 2 and 4H (2H vs. 4H, q = 0.0034), 2H and 12H (2H vs. 12H, q = 0.0012), 2H and 24H (2H vs. 24H, q = 0.0064). We observed concomitant changes in numbers of activated microglia (FC, F (5,12.85) = 68.28, *p* < 0.0001). Post-hoc analyses of the numbers of activated microglia in the frontal cortex showed significantly more activated microglia in every HI group (2H, 236 ± 45; 4H, 263 ± 17; 8H, 281 ± 17; 12H, 287 ± 24; 24H, 258 ± 27) compared with the control group (27 ± 4, q < 0.001 for all comparisons). Similarly significant differences in the numbers of resting microglia were found in the putamen (Fig. 2 O; PUT, (F (5, 6.358) = 254.2, *p* < 0.0001; 2H, 20 ± 11; 4H, 13 ± 8; 8H, 9 ± 5; 12H, 10 ± 4; 24H, 6 ± 6) compared with the control group (264 ± 31, q < 0.001 for all comparisons). Similarly, there were significant differences in the number of activated microglia in the putamen (PUT, F (5, 11.47) = 69.51, *p* < 0.0001). Analysis of the numbers of activated microglia in the putamen showed the control group (25 ± 4) had significantly fewer activated microglia than every other group (2H, 235 ± 47; 4H, 261 ± 17; 8H, 286 ± 25; 12H, 287 ± 16; 24H, 255 ± 24) (C vs. 2H, q < 0.0001; C vs. 4H, q < 0.0001; 8H q < 0.0001; 12H q < 0.0001; 24H, q < 0.0001). There were significant differences in the total number of Iba-1 + cells in the frontal cortex (Fig. 2*P*; F (5,22) = 2.888, *p* = 0.0375). Post-hoc analyses did not reveal significant differences between specific HI groups. No significant differences in the total number of Iba-1 (F (5,22) = 2.555, *p* = 0.0573) were observed in the putamen.

### Hypoxic-ischemic injury is associated with changes in astrocyte morphology and reduced astrocyte coverage

**Figure 3:**
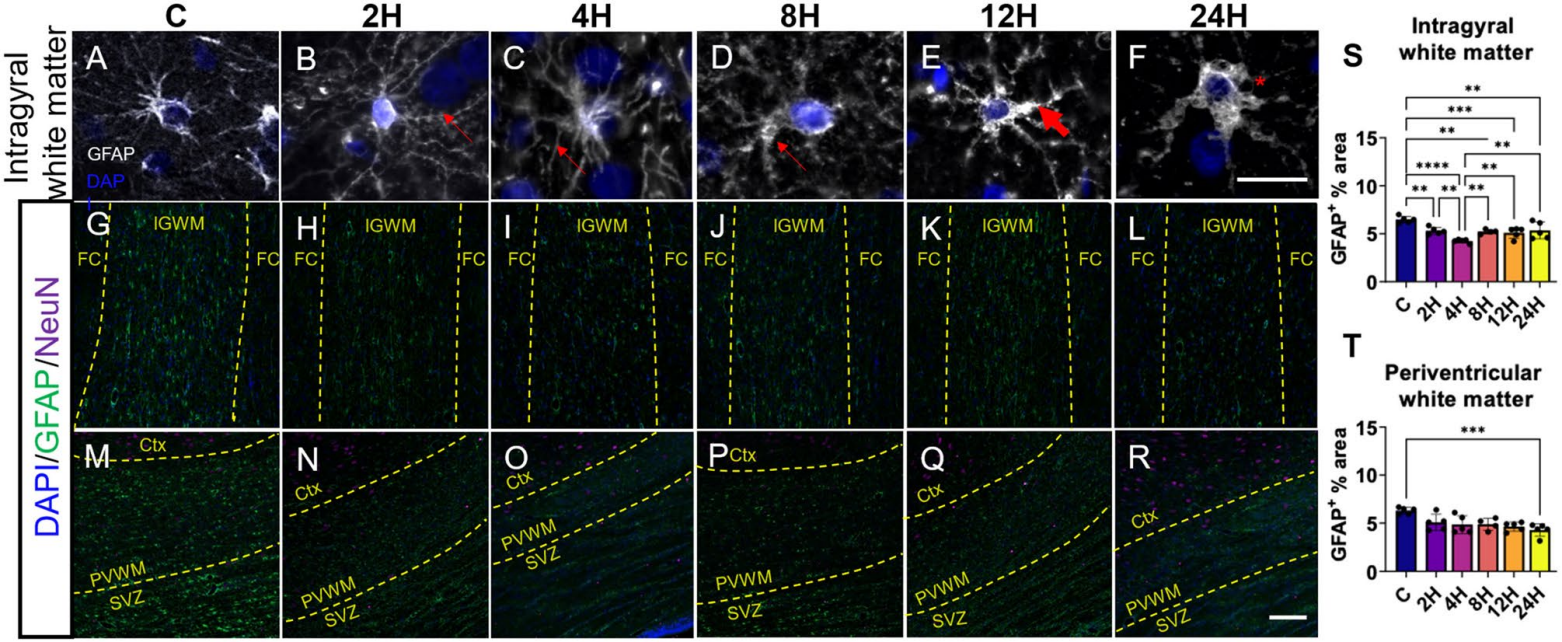
Hypoxic-ischemic injury is associated with significant alterations to astroglial coverage accompanied by changes in astrocyte morphology. Representative image of astrocytes labelled with glial fibrillary acidic protein (GFAP). (**A**) Astrocytes in C animals displayed long extended processes and small cell bodies, typical of resting astrocytes. (**B**, **C**) In the first 4H following insult, the cell bodies of the astrocytes began to hypertrophy, with gradual loss of the fine tertiary processes. (**D**, **E**) Between 4-8H, the primary processes began to thicken, followed by a distinct reorganization of the secondary processes seen at 12H. (**F**) The 24H group had few secondary processes, profoundly hypertrophied primary processes, and cell bodies. The scale bar equals 20 μm (**A**–**F**). Representative images of astrocyte coverage (GFAP) in the intragyral (**G**–**L**) and paraventricular (**M**–**R**) white matter regions. The scale bar indicates 250 μm (**G**–**R**). Quantification of GFAP expression using densitometry showed a decrease in GFAP expression in 2H, 4H, 12H and 24H groups in the intragyral white matter (**S**), and 24H group in the periventricular white matter (**T**). Column graphs illustrate the mean with error bars at one standard deviation. Statistical comparisons refer to ordinary one-way analysis of variance test with a two-stage linear step-up procedure of Benjamini, Krieger and Yekutieli. Statistical significance was accepted at q < 0.05 (^∗^q < 0.05, ^∗∗^q < 0.01, ^∗∗∗^q < 0.001, ^∗∗∗∗^q < 0.0001). *C* control group; *Ctx* cortex; *DAPI’ 4′*6-diamidino-2-phenylindole; FC, frontal cortex; GFAP, glial fibrillary acidic protein; (number)H, group culled hours post injury; IGWM, intragyral white matter; NeuN, neuronal nuclei; PVWM, periventricular white matter.

Like microglia, astrocytes have been shown to respond to HI injury with morphological changes. GFAP was used to label astrocytes in the piglet brain (Fig. 3). Astrocytes in the control group had small cell bodies and branching pattern typical of healthy astrocytes (Fig. 3A) The 2H and the 4H groups showed enlarged astrocytic cell bodies, with gradual loss of the fine tertiary processes (Fig. 3B,C). Between 4 and 8H the primary processes began to thicken (indicated by thin arrows), followed by distinct reorganization of the secondary processes in the 12H group (Fig. 3D,E, indicated by thick arrows). The 24H group had few secondary processes, and profoundly hypertrophied primary processes and cell bodies (Fig. 3F, indicated by asterisk). Representative fields of view demonstrate a reduction in coverage in both the intragyral white matter (Fig. 3G–L) and the periventricular white matter (Fig. 3M–R). Quantification of the astrocyte coverage showed a significant reduction in coverage between groups in the white matter regions (Fig. 3S; IGWM, F (5,23) = 10.36, *p* = 0.0001; Fig. 3T; PVWM, F (5,23) = 5.199, *p* = 0.0025). Post-hoc analyses of GFAP coverage in the IGWM found significant reduction in all HI groups, (2H, 5.207 ± 0.4415; 4H, 4.239 ± 0.1981; 8H, 4.915 ± 0.5983; 12H, 5.009 ± 0.600; 24H, 5.345 ± 0.8724) compared with control group (6.471 ± 0.341, q < 0.01 for all comparisons). Furthermore, significant differences were observed between 2 and 4H (2H vs. 4H; q = 0.0023), 4H and 8H (4H vs. 8H; q = 0.0054), 4H and 12H (4H and 12H; q = 0.0083), 4H and 24H (4H vs. 24H; q = 0.0019). Post-hoc analyses of GFAP coverage in the PVWM found that all groups (2H, 4.859 ± 0.9303; 4H, 4.857 ± 0.923; 8H, 4.866 ± 0.607; 12H, 4.530 ± 0.4432; 24H, 4.282 ± 0.6531) were significantly reduced compared with the control group (6.297 ± 0.331; q < 0.02 for all comparisons).

### Hypoxic-ischemic injury is associated with increased mRNA expression of pro-inflammatory cytokines

**Figure 4:**
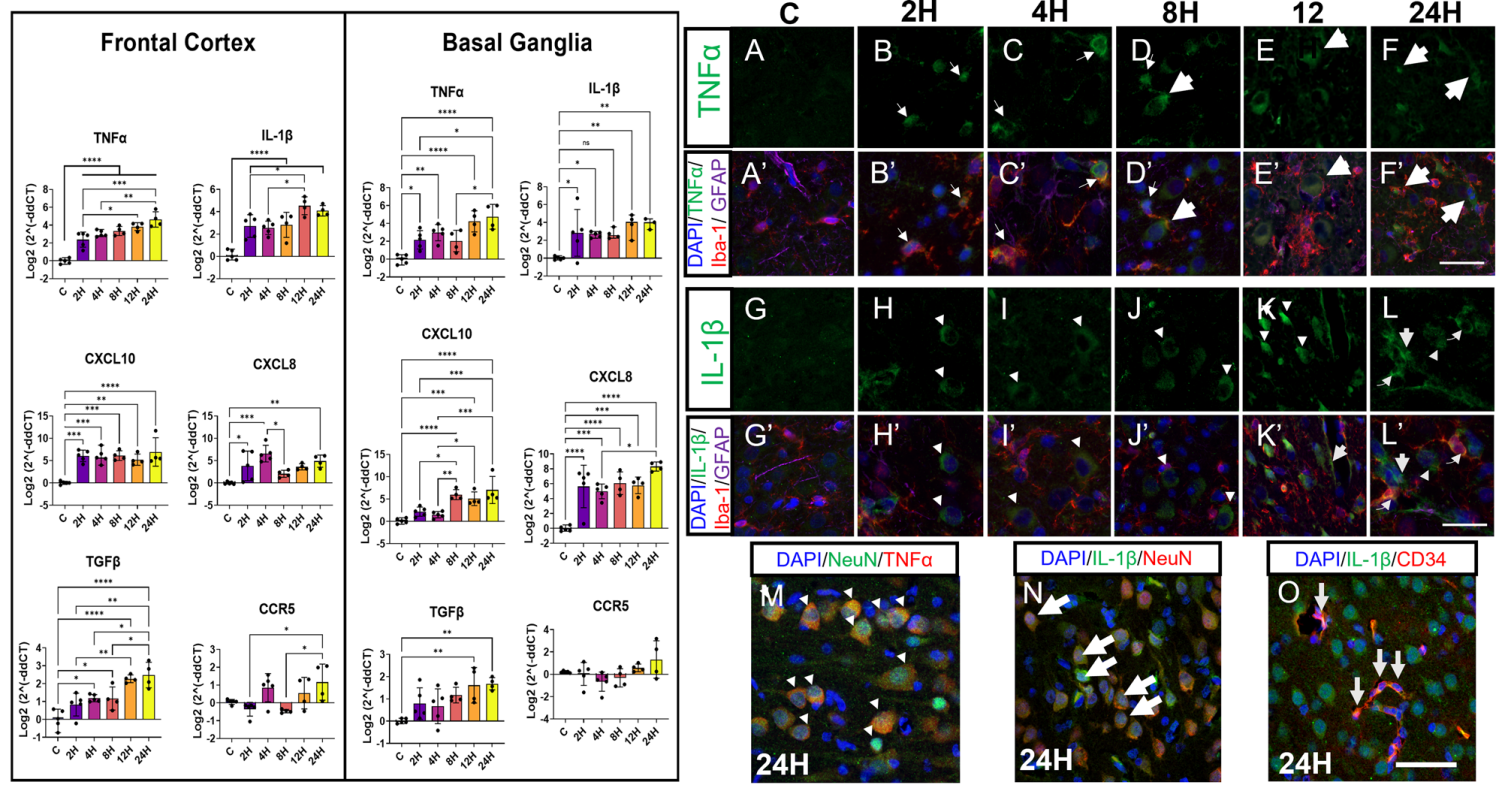
Inflammatory related mRNA changes in the parasagittal frontal cortex (left) and the basal ganglia following HI insult. Results of multiple comparisons are indicated on the column graphs. Colocalization studies were performed for cytokines tumor necrosis factor alpha (TNFα) and interleukin (IL-1β). Representative images of TNFα labelling (**A**–**E**). TNFα was colocalized with microglia (Iba-1) in 2H and 4H groups (A’, B’; thin tailed arrows). In the 8H and 12H groups, TNFα was frequently colocalized with microglia (C’, D’; thin tailed arrows) and cells resembling neurons (D; large thick-tailed arrows). TNFα expression was lost in the microglia and was expressed mainly by cells resembling neurons in the 12H and 24H groups (D’, E’; large thick-tailed arrows). Representative labelling of IL-1β (**F**–**J**). IL-1β was associated with cells resembling neurons in 2H-8H group (F’-H’; triangles). In the 12H group, there was possible neuronal and vascular staining (I’; small thick-tailed arrows). 24H group showed colocalization of IL-1β with possible neurons (triangle), vascular structures (thick tailed arrow), and microglia (curly arrow) (J’). To confirm the association of TNFα with neurons, TNFα was colocalised with NeuN (M; arrow heads). Similarly, IL-1β was IL-1β was colocalized with NeuN and CD34, confirming the association between IL-1β and both neurons (thick tailed arrows) and vascular structures (thin tailed arrows; N,O). Scale bar is equal to 25 μm (A–L’). Scale bar is equal to 25 μm (**M**–**O**). Individual data points indicate the average aggregation of 3 technical replicates. Columns illustrate the mean with error bars at one standard deviation. Statistical comparisons refer to ordinary one-way analysis of variance test with a two-stage linear step-up procedure of Benjamini, Krieger and Yekutieli. Statistical significance was accepted at q < 0.05 (^∗^q < 0.05, ^∗∗^q < 0.01, ^∗∗∗^q < 0.001, ^∗∗∗∗^q < 0.0001). Abbreviations: CD-34, cluster of differentiation- 34; CCR5, C–C chemokine receptor type 5; CXCL8, C-X-C motif chemokine ligand 8; CXCL10, C-X-C motif chemokine ligand 10; DAPI’ 4',6-diamidino-2-phenylindole; GFAP, glial fibrillary acidic protein; (number)H, group culled hours post injury; Iba-1, ionized calcium binding adaptor molecule 1; IL1β, interleukin-1 beta; TNFα, tumour necrosis factor alpha; TGFβ, transforming growth factor.

Given the level of glial cell activation seen in the HI groups, it was hypothesized that this would be associated with an increased expression of inflammation-related markers. qPCR and immunofluorescence was used to assess the effect of HI on the expression of inflammation-related molecules (Fig. 4). In the frontal cortex, TNFα, IL-1β, and CXCL10 were significantly upregulated at every time point compared with control animals. CXCL8 levels were significantly different at all timepoints except in the 8H group. TGF-β was upregulated in 4H, 8H, 12H and 24H groups. No significant differences were observed in the levels of CCR5 between Controls and HI groups at any time point. Similarly, in the putamen, levels of TNFα, IL-1β and CXCL8 were upregulated in every HI group compared with control levels. CXCL10 and TGF-β was upregulated in the 8H, 12H and 24H group compared with controls. No significant differences were observed in levels of CCR5 in either region of interest.

Immunofluorescent labelling was used to qualitatively determine the cellular localization of TNFα and IL-1β in the frontal cortex. There was no detectable expression of TNFα or IL-1β in C brains (Fig. 4A,A’, G,G’). In the HI injured animals, the overall the staining patterns of both markers were focal to small regions of the tissue area analysed. In these foci, TNFα colocalized only with microglia (Iba-1) in 2H and 4H groups (thin tailed arrows; Fig. 4B,C). In the 8H group, TNFα was frequently colocalized with microglia (thin tailed arrows) and cells of neuronal morphology (Fig. 4D,D’; large thick-tailed arrows). TNFα expression was lost in the microglia and was expressed mainly by cells of neuronal morphology in the 12H and 24H group (Fig. 4E–F’; large thick-tailed arrows). IL-1β colabelled with cells of neuronal morphology in the 2H, 4H and 8H groups (Fig. 4H–J’; arrowheads). In the 12H group, there was labelling of cells of neuronal morphology and vascular labelling (Fig. 4K’; small thick-tailed arrows). Animals in the 24H group showed labelling of cells of neuronal morphology (arrow head), vascular (large thick tailed arrows), and microglial (curly arrow) IL-1β labelling (Fig. 4L’). To confirm the association of TNFα and IL-1β with neurons and vascular structures, additional colocalization with NeuN and CD-34 was performed in a subset of 24H animals. This vascular and neuronal labelling with TNFα and IL-1β was confirmed by co-labelling with NeuN and with cluster of differentiation (CD)-34 (Fig. 4M–O).

### Global hypoxic-ischemic injury was associated with neuronal death

**Figure 5:**
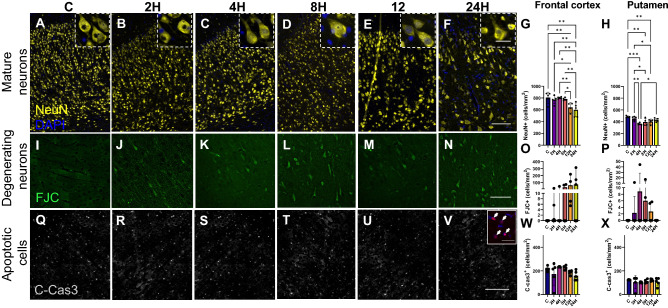
Hypoxic-ischemic injury was associated with alterations in NeuN positive cells, but not numbers of degenerating neurons or possibly apoptotic cells. Immunofluorescent staining of mature neurons using the neuron-specific nuclear marker NeuN. All piglet brains displayed robust NeuN expression throughout the frontal cortex (FC) (**A**–**F**). Quantification of NeuN showed significant differences in the number of mature in both the FC (**G**) and PUT (**H**). There were significantly fewer mature neurons in the 12H and 24H in the FC and significantly fewer mature neurons in the 4H, 8H and 12H groups in the PUT compared with C control brains. Column graphs illustrate the mean with standard deviation indicated by the error bars. FJC-positive cells displayed distinct staining of cell bodies and processes (**I**–**N**). Column graphs illustrate the median with 95% confidence intervals. No significant difference.

**Figure 6:**
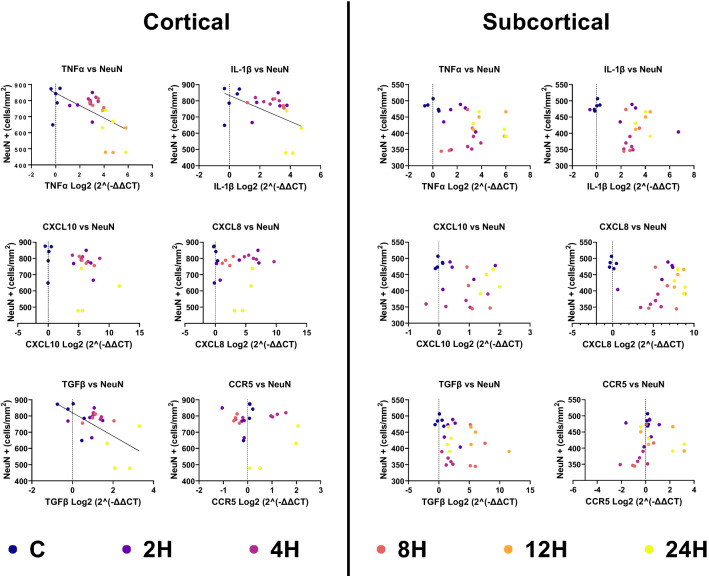
The numbers of NeuN positive cells were inversely correlated with levels of TNFα, IL-1β, and TGFβ. Statistical refer to the results of a pearson correlation test. Statistical significance was accepted at *p* < 0.05. Abbreviations: CD-34, cluster of differentiation- 34; CCR5, C–C chemokine receptor type 5; CXCL8, C-X-C motif chemokine ligand 8; CXCL10, C-X-C motif chemokine ligand 10; DAPI, 4’,6-diamidino-2-phenylindole; GFAP, glial fibrillary acidic protein; (number)H, group culled hours post injury; Iba-1, ionized calcium binding adaptor molecule 1; IL1β, interleukin-1 beta; TGFβ, transforming growth factor βTNFα, tumour necrosis factor alpha; TGFβ, transforming growth factor β.

With the preponderance of glial cell activation and inflammation-related gene transcription, it was hypothesized that this would be associated with a reduction in the number of mature neurons. NeuN was used to visualize the neuronal soma in the frontal cortex and the putamen. Figure 5A–F shows robust staining of the neuronal cell bodies in the frontal cortex. In total, 81111 NeuN + cells were counted in the frontal cortex and 64984 NeuN + cells in the putamen. A significant difference was found in the frontal cortex (Fig. 5G) [F (5,22) = 6.390, *p* = 0.0008], and the putamen [F (5,22) = 7.351, *p* = 0.0003] (Fig. 5H). Multiple comparisons against the control group (805 ± 95) showed that the 12H (621 ± 83) and 24H (600 ± 117) groups had significantly fewer NeuN + cells [C vs. 12H_,_ q = 0.0027; C vs 24H, q = 0.0020] in the frontal cortex. Significant differences were also observed across time between 2 and 12H (2H vs 12H; q = 0.0187), 2H and 24H (2H vs. 24H; q = 0.0037), 4H and 12H (4H vs. 12H; q = 0.0062), 4H and 24H (4H vs. 24H; q = 0.0037), 8H and 12H (8H vs. 12H; q = 0.0172), 8H and 24H (8H vs. 24H; q = 0.0037). In the putamen, multiple comparisons against the control group (484 ± 15) showed that the 4H (364 ± 17), 8H (395 ± 62), 12H (403 ± 44), and 24H (430 ± 30) group had significantly fewer NeuN + cells [C vs. 4H, q = 0.0002; C vs. 8H, q = 0.0037; C vs.12H, q = 0.0060; C vs. 24H, q = 0.0031]. 2H brains had significantly more NeuN + cells than 4H (2H vs. 4H; q = 0.0023), 8H (2H vs. 8H; q = 0.0293), and12H (q = 0.0438). 4H was significantly more than 24H (q = 0.0142). FJC was used to identify degenerating neurons (Fig. 5 I–N). In total, 2979 FJC + cells were counted in the FC, and 1892 were counted in the PUT. Results exhibited high interindividual and intraindividual variability, with strong positive skews and a high degree of heteroscedasticity. No significant differences were detected in either the frontal cortex (Fig. 5O; χ^2^ (6) = 10.50, *p* = 0.0623), or the putamen (Fig. 5P; χ^2^ (6) = 6.188, *p* = 0.2933). Cleaved-caspase-3 was used to identify potentially apoptotic cells (Fig. 5Q–V). In total 34629 caspase 3 + cells were quantified in the frontal cortex and 24711 in the putamen. No significant differences were detected in either the frontal cortex [Fig. 5W; F (5,22) = 1.351, *p* = 0.2804], or the putamen [Fig. 5X; F (5,22) = 1.092, *p* = 0.791]. Both analyses failed the Shapiro-Wilks test for normality due to a few outliers. Given that one-way ANOVA has been demonstrated to be robust against violations of normality with similar group size, skewness and kurtosis^28^, these data were not reanalyzed with Kruskal–Wallis tests. To provide further evidence of an association of neuronal cell death and inflammation, Pearson correlation analyses were conducted between neuronal cell counts in the frontal cortex and measures of inflammation related gene transcription in the frontal cortex, and neuronal cell counts in the putamen with inflammation related gene transcription in the basal ganglia (Fig. 6). Significant correlations were observed in the frontal cortex between the number of NeuN positive cells and TNFα (R^2^ = 0.3402, *p* = 0.0014; y = − 38.53*x* + 845.6), NeuN and IL-1β (R^2^ = 0.244, *p* = 0.0088; *y* = − 40.15*x* + 833.4), and CXCL10 (R^2^ = 0.355, *p* = 0.001; *y* = − 71.62*x* + 820.2). No significant correlations were observed between neuronal cell numbers in the PUT and gene transcription in the basal ganglia was observed in the numbers of FJC + cells in both the FC (O) and the PUT(P). Column graphs illustrate the mean with standard deviation indicated by error bars. Representative image of possibly apoptotic cells (cleaved-caspase 3 + cells, grey/red) in the cortex of all piglets (Q-V). Inset clearly shows colocalisation with DAPI indicating + staining (scale bar = 200 μm, inset scale bar = 50 μm). No significant differences in the number of C-cas3 cells were observed in either the FC(W) or the PUT(X) Statistical comparisons for NeuN and C-cas3 labelling refer to ordinary-one way analysis of variance with a two-stage linear step-up procedure of Benjamini, Krieger and Yekutieli. Statistical comparisons for FJC labelling refer to the Kruskal–Wallis test with a two-stage linear step-up procedure of Benjamini, Krieger and Yekutieli. Statistical significance was accepted at q < 0.05 (^∗^q < 0.05, ^∗∗^q < 0.01, ^∗∗∗^q < 0.001, ^∗∗∗∗^q < 0.0001). Abbreviations: C-cas3, cleaved-caspase 3; CI, confidence interval; GFAP, glial fibrillary acidic protein; (number)H, group culled hours post injury; HIE, hypoxic-ischemic encephalopathy; Iba-1, ionized calcium binding adaptor molecule 1; NeuN, neuronal nuclei.

## Discussion

This study examined the evolution of neuroinflammation in our piglet model of HIE. Differences in glial cell morphology in HI piglets were observed at several time-points, suggesting increased neuroinflammation in HI injured piglets. These changes were associated with changes in the expression of inflammation-related molecules measured via qPCR. The source of TNFα was found to change from microglia in the early time-points (2H–8H) to neurons (8H–24H), and the source of IL-1β expression changed from neurons in the early time-points (2H–12H) to expression by vascular structures (12H, 24H) and microglia (24H). These data strongly indicate a pro-inflammatory state in the frontal cortical region, though there was less evidence of this in the putamen. Furthermore, this pro-inflammatory state was associated with decreases in the number of NeuN positive cells, but not the number of degenerating neurons or potentially apoptotic cells. Lastly, TNFα, IL-1β and TGFβ were found to be inversely related to the number of NeuN positive cells.

Increased activated microglia and corresponding decreased resting microglia following neonatal HI is considered a hallmark of HIE neuropathology^29,30^. These results are consistent across mammalian species, with studies showing a neuroinflammatory response in the HI injured brain with increased microglial activation and astrogliosis, though the temporal dynamics of this have rarely been examined.

In this study, significant upregulation of activated microglia was observed at every time point, with a corresponding reduction in resting microglia. Changes were detected in microglial phenotype at 2H post-injury, but the earliest time point at which microglia began to change in our model is unknown. Ivacko et al.^30^ made the seminal observations of microglia morphology in a model of neonatal HIE (P7 rat). They noted subtle morphologic evidence of microglial activation as early as 10 min post-HI in the lesioned right cerebral hemisphere, and activated microglia began to accumulate within the next 4 h. Accumulation of activated microglia peaked at 2–4 days post-HI^30^. In our study, while the numbers of Iba-1 + cells differed significantly compared with controls, we also observed no difference between HI timepoints. However, our model differs significantly, as the ischaemic component is reversible and comparatively less severe than rodent models.

Glial fibrillary acidic protein (GFAP) is an intermediate filament protein that modulates astrocyte motility and shape, providing structural stability to processes^31^. Previous studies^11,12^ from our laboratory have examined the morphology of astrocytes at 8 and 72 h post insult. In our earlier studies, brain regions without significant neuronal damage showed minimal perturbation to astrocyte morphology. However, astrocytes in damaged regions of HI brains exhibited altered morphologies dependent on the degree of injury. These ranged from mild thickening of processes to astrocytes with multiple shorter primary processes lacking secondary or tertiary branching (like those seen in the 2H group) or exhibited abnormal features such as thickening of the processes (like those seen in 8H, 12H and 24H groups) and terminal swellings. Furthermore, our previous work showed decreased GFAP coverage by approximately 30% in the white matter regions following HI; in the current study, we observed approximately 25% reduction in GFAP coverage. Martin et al.^32^ reported a decrease in GFAP-positive cell body densities at 24- and 48-h post-insult, indicating astrocyte death, with recovery of numbers by 96 h post-insult. It may be that the initial reduction in GFAP expression seen in our study might be an early phase response to HI. It would have been valuable to extend this research to include animals culled at 48, 72 and 96 h to understand whether astrocyte expression rebounds in our model.

Increased expression of TNFα and IL-1β mRNA at 2H, 4H, 8H, 12H and 24H post- HI insult was observed. Previous studies in P7 rats showed changes in IL-1β mRNA with the highest expression at 3 h after HI and highest IL-1β bioactivity at 6 h^33^. Szaflarski et al.^34^ in the same model, reported a maximum expression of these cytokines at 4 h. Moreover, perinatal brain injury elicited by focal intracerebral N-methyl-d-aspartate injection showed a corresponding acute stimulation of IL-1β and TNFα mRNA expression 4 h after injection^34^. Bonestroo et al.^35^ in P7 Wistar rats reported an upregulation of the pro-inflammatory cytokines TNFα and IL-1β and anti-inflammatory IL-10 mRNA levels at 3 h post-injury compared with sham-operated animals. Twenty-four hours after insult, an increase in macrophages/microglia was observed around the cerebral lesion as well as an increase in granulocyte influx compared to 3 h after HI^35^.

In contrast to the rapid maximal expression of cytokines demonstrated in previous rodent studies, our study showed the highest expression of inflammatory cytokines between 8 and 24 h, and the direction of the trends suggest that the inflammatory state is increasing in magnitude over time. Differences in the peak cytokine expression may be due to the differences between the piglet and rodent animal models. In the rodent experiments above, the ischemic component is induced by carotid artery occlusion and is irreversible, producing total infarction, regions of massive cell loss and brain atrophy, unlike the reversible hypotensive ischaemia in the piglet which produces selective neuronal cell loss in the watershed regions. Thus differences in the mode of induced damage may account for the differences in the evolution of inflammation.

Other inflammatory genes that have been associated with immune cell infiltration include CCR5 and CXCL8^36^. In this study, CCR5 mRNA expression was not significantly different compared with controls. However, CXCL8 is rapidly upregulated and remained elevated for at least 24 h post-insult in the putamen, and has a biphasic pattern of expression in the frontal cortex. CXCL8 has previously been implicated in enhancing neutrophil infiltration across the BBB in adult stroke models^36^. However, neutrophil transmigration occurs at lower rates in neonates than adults following HI^37^; therefore, it is unclear what the impact of CXCL8 upregulation is in our model.

There are few investigations of CXCL10 in neonatal HI models. In one study in the P7 neonatal rat, CXCL10 was substantially upregulated in HI animals 3-, 6-, and 24 h post-HI compared with the control group^38^. Donega et al.^39^ demonstrated that CXCL10 was upregulated in the ischemic penumbra 10 days post-insult and downregulated 17 days post-insult in a neonatal mouse model^39^. These studies agree with our data, showing CXCL10 expression is significantly and substantially upregulated at every time point studied in the frontal cortex, and at 8H, 12H and 24H in the putamen. The results of Donega et al. suggest this upregulation may be prolonged out to 10 days, though this should be experimentally determined due to the differences already discussed between animal models.

TGFβ is commonly categorized as an anti-inflammatory cytokine, and its expression is thought to contribute to resolution rather than injury^40^. However, this notion is challenged by the observation that inhibition of the TGFβ receptor has been repeatedly observed to reduce neurodevelopmental deficits, improve cognitive function, and reduce pathological astrogliosis^41,42^ following neonatal HI injury. In addition, TGFβ has previously been shown to be upregulated in neonates with moderate HIE at 24 h, one week, and one month post-birth^43^. Given the substantial upregulations of TGFβ mRNA expression demonstrated in this study, there is a need for a systematic investigation into the role of TGFβ expression and pathway signalling in HIE.

The activation of microglia and reactive astrocytes produces pro-inflammatory cytokines such as TNFα and IL-1β that are toxic to neurons. This study shows that neurons and the vasculature are also significant sources of these pro-inflammatory cytokines in HI injured piglet brains. TNFα was expressed in microglia in the early hours (2H, 4H) following injury before being predominantly expressed in neurons in the later time-points (8H–24H). Janelsins et al.^44^ demonstrated that selective neuronal expression of TNFα in rodent brain resulted in microglial activation and upregulation of downstream target molecules that leads to peripheral cell infiltration and exacerbation of the neuroinflammatory state^44^. So, it is possible that the expression we see in neurons is pathological.

Furthermore, neuronal IL-1β was detected in the HI injured brain at 2H–8H post insult, with vascular involvement at 12H and microglial involvement at 24H with no detectable expression in astrocytes in the frontal cortex. Savard et al.^45^, in a rodent model of neonatal HIE, neurons were the first cells to produce excessive levels of IL-1β. Further studies showed IL-1β plays a significant role in neuronal self-injury through matrix metalloproteinase (MMP)-9^46^. The demonstration here that cellular sources of cytokines change dynamically throughout the injury period may indicate that the physiological effect of these molecules may be different at different time points, and classifying these molecules as pro-inflammatory or anti-inflammatory may be a misleading oversimplification.

Surprisingly, despite a reduction in the number of NeuN positive cells, no alterations in FJC or C-cas3 were observed in either brain region. Firstly, loss of NeuN immunoreactivity has previously been shown to be reduced following cerebral ischemia, without destruction of the cells^47^. Secondly, all groups except frontal cortex 8H contain multiple instances of 0 FJC positive cells, we believe this is what is driving the lack of statistical significance. The mechanism of FJC is unknown, though it is believed to be specific to degenerating neurons regardless of the mechanism of cell death (i.e. necrosis, apoptosis, autophagy etc.)^48^.Though some have shown degenerating microglia, astrocytes, and mesenchymal cells can be FJC positive under some conditions in mice^49^, so it is possible that our cell counts may included degenerating cells of other cell types.

Some studies have shown that C-cas3 is a poor marker of cell death/apoptosis and may reflect caspase's non-apoptotic functions^50,51^. Acarin et al.^52^ analysed astrocyte cleavage of caspase-3 following excitotoxic damage in postnatal rats to determine if its presence is associated with apoptotic cell death, cell proliferation, or cytoskeletal remodelling. They showed C-cas3 was mainly observed in the nucleus of activated astrocytes in the lesioned hemisphere as early as 4 h post injury and persisted until the glial scar was formed at 7–14 days, and importantly, it was not associated with TUNEL labelling. Furthermore, astrocyte caspase-3 cleavage was not associated with dividing cells. They suggested that caspase activation is critical for astrocyte cytoskeleton remodelling following cellular injury^52^. Others in a similar HI piglet model have previously observed discrepancies between TUNEL-positive cell death and C-cas3^53,54^. Thus, supporting the proposition that much of the C-cas3 observed in this model is not a part of the apoptotic cascade but instead may reflect astrocyte remodelling, and should be interpreted with caution, and should be taken into account when planning similar studies in the future.

Several limitations should be acknowledged. First, comparisons were made against a healthy control sample population rather than a sham-operated sample population. This is important because several actions in our experimental setup are known to induce changes in glial cell activation, including exposure to isoflurane^55,56^, heparin^57^, and mild hypoxia^58^ due to delayed intubation, all of which are not recorded in our protocols and thus could not be controlled for statistically. Second, morphological classification of microglia is an inherently subjective technique. Some automated tools for classifying microglia have been developed and may be used in future studies^59,60^. Finally, the number of animals in each group to was relatively low (n = 4–5); however, we found the effect size to be considerable demonstrating significant differences between the groups.

## Conclusions

This is the first study to chronicle the change in brain inflammation following global HI injury in a large animal model. There was a significant increase in inflammatory cytokines and chemokines from 2H following HI insult. This was associated with glial cell activation in all HI injured groups with significant loss of NeuN positive cells observed at 4H in the putamen and 8H in the frontal cortex, and no significant changes to the numbers of potentially apoptotic cells. Furthermore we demonstrated that the cellular localisation of proinflammatory cytokines IL-1β and TNFα differs over time, highlighting the cellular complexity of the inflammation cascade. Current clinical guidelines state that hypothermia therapy be initiated as soon as possible following resuscitation, between 1 and 6 h of life. This data underscores the importance of initiating treatment as soon as practicable following injury, to limit the inflammatory load and the consequential effect on neighbouring neuronal populations.

## Supplementary Information

Supplementary Information 1.

Supplementary Information 2.

## Data Availability

All data generated or analyzed during this study are included in this published article and its supplementary information files.

## Abbreviations

C: Control group
CCR5: C–C chemokine receptor type 5
C-cas3: Cleaved-caspase 3
CI: Confidence interval
CXCL8: C-X-C motif chemokine ligand 8
CXCL10: C-X-C motif chemokine ligand 10
GFAP: Glial fibrillary acidic protein
HIE: Hypoxic-ischemic encephalopathy
Iba-1: Ionized calcium binding adaptor molecule 1
IL-1β: Interleukin 1 beta
i.v: intravenous
MABP: Mean arterial blood pressure
NeuN: Neuronal nuclei
P(number): Postnatal day
PBS: Phosphate buffered saline
pCO_2_: Partial pressure of carbon dioxide
pO_2_: Partial pressure of oxygen
qPCR: Quantitative polymerase chain reaction
RT: Room temperature
TGFβ: Transforming growth factor beta
TNFα: Tumor necrosis factor alpha
