# Supplementary material for: Early evolution of glial morphology and inflammatory cytokines following hypoxic-ischemic injury in the newborn piglet brain
Source: Sci Rep. 2023 Jan 6;13:282. doi: 10.1038/s41598-022-27034-9 (PMC9823001; doi:10.1038/s41598-022-27034-9)
Supplement: Supplementary file 1 — Supplementary Information 1. [file 41598_2022_27034_MOESM1_ESM.docx]

Table 1. Primary antibodies used in this study with specific incubation conditions. Abbreviations: ON, overnight; RT, Room temperature.

| **Primary antibody** | **Host** | **Incubation conditions**  **(Dilution, incubation time, temperature)** | **Marker** | **Catalogue #** |
| --- | --- | --- | --- | --- |
| Cleaved-caspase 3 (C-Cas3) | Goat | 1:500, ON at RT | Apoptotic cell marker | Cell Signalling (#9661) |
| Cluster of differentiation(CD)-34 | Goat | 1:1000,ON at RT | Endothelial progenitor cell marker | R&D Systems (AF3890) |
| Glial fibrillary acidic protein (GFAP) | Mouse | 1:1000, ON at RT | Astrocyte marker | Sigma (G3893) |
| Glial fibrillary acidic protein (GFAP) | Rabbit | 1:1000, ON at RT | Astrocyte marker | DAKO (Z0334) |
| Ionised calcium binding adaptor molecule1 (Iba-1) | Goat | 1:1000, ON at RT | Pan -microglial marker | Abcam (ab5076) |
| Ionised calcium binding adaptor molecule1 (Iba-1) | Rabbit | 1:1000, ON at RT | Pan -microglial marker | Abcam (ab178846) |
| Interleukin (IL)-1β | Rabbit | 1:100, ON at 4°C | Inflammatory marker | Abcam (ab9722) |
| Neuronal Nuclei (NeuN) | Rabbit | 1:1000, ON at RT | Neuronal marker | Abcam (ab177487) |
| Tumour necrosis factor (TNF)α | Goat | 1:200, ON at 4°C | Inflammatory marker | R&D Systems (AF-410) |

Table 2: Secondary antibodies and incubation conditions used in this thesis. Abbreviations: RT, room temperature.

| **Secondary antibody** | **Host** | **Incubation (dilution, time (h), temperature)** | **Catalogue #** |
| --- | --- | --- | --- |
| α-Rabbit Alexa fluor 594 | Donkey | 1:1000, 1 hour at RT | Invitrogen (A-21447) |
| α-Rabbit Alexa fluor 488 | Donkey | 1:1000, 1 hour at RT | Invitrogen (A-11057) |
| α-Mouse Alexa fluor 647 | Donkey | 1:1000, 1 hour at RT | Invitrogen (D1306) |
| α-Mouse Alexa fluor 568 | Donkey | 1:1000, 1 hour at RT | Invitrogen (A-21207) |
| α-Goat Alexa fluor 647 | Donkey | 1:1000, 1 hour at RT | Invitrogen (A-31571) |
| α-Goat Alexa fluor 568 | Donkey | 1:1000, 1 hour at RT | Invitrogen (A10037) |
|  |  |  |  |
| DAPI (4',6-Diamidino-2-Phenylindole, Dihydrochloride) | - | 1:2000, 1 hour at RT | Invitrogen (D1306) |

Table 3: Gene specific primer characteristics. Abbreviations: F, forward primer; R, reverse primer.

| ***Gene name*** | ***Primer sequence (F, R)*** | ***Relevance*** | ***Product length*** | ***Annealing Temp*** | ***Accession number*** |
| --- | --- | --- | --- | --- | --- |
| *CCR5* | *TGGTCAGAGGAGCTGAGACA* | Chemokine | *86* | *60* | *NM_001001618.1* |
|  | *AGAAGGGACTCGTCGTTTGA* |  |  |  |  |
| *CXCL10* | *CCCACATGTTGAGATCATTGC* | Chemokine | *168* | *62.3* | *NM_001008691.1* |
|  | *CATCCTTATCAGTAGTGCCG* |  |  |  |  |
| *GAPDH* | CTTCACGACCATGGAGAAGG | Reference gene | *170* | *62.7* | *XM_003361568.3* |
|  | *CCAAGCAGTTGGTGGTGCAG* |  |  |  |  |
| *IL-1β* | *AGAAGAGCCCATCGTCCTTG* | Cytokine | *70* | *58.4* | *NM_214055.1* |
|  | *GAGAGCCTTCAGCTCATGTG* |  |  |  |  |
| *CXCL8* | *GAAGAGAACTGAGAAGCAACAACA* | Cytokine | *99* | *58.4* | *NM_213867.1* |
|  | *TTGTGTTGGCATCTTTACTGAGA* |  |  |  |  |
| *TGFβ* | *CGAGCCCTGGATACCAACT* | Cytokine | *135* | *62.7* | *NM_214015.2* |
|  | *GCAGAAATTGGCATGGTAG* |  |  |  |  |
| *TNFα* | *CCAATGGCAGAGTGGGTATG* | Cytokine | *116* | *62.7* | *NM_214022.1* |
|  | *TGAAGAGGACCTGGGAGTAG* |  |  |  |  |
